# Shaping Efficiency of Heat-Treated Reciprocating Systems in Mandibular Molars: A Micro-CT Analysis

**DOI:** 10.1055/s-0045-1812495

**Published:** 2025-12-18

**Authors:** Thamires Custódio Matos, Rayana Duarte Khoury, Carlos Henrique Sales Dias Santos, Ana Flávia Barbosa, Ricardo Tadeu Lopes, Emmanuel João Nogueira Leal Silva, Ana Paula Martins Gomes

**Affiliations:** 1 Department of Restorative Dentistry, São Paulo State University (UNESP), Institute of Science and Technology, São José dos Campos, SP, Brazil; 2 Department of Endodontics, Rio de Janeiro State University, Rio de Janeiro, Brazil; 3 Nuclear Engineering Program, Federal University of Rio de Janeiro (UFRJ), Rio de Janeiro, Brazil; 4 Department of Endodontics, School of Dentistry, Grande Rio University (UNIGRANRIO), Rio de Janeiro, Brazil

**Keywords:** dental instruments, endodontics, heat treatment, micro-computed tomography, root canal preparation, root canal therapy

## Abstract

**Objectives:**

This study aimed to evaluate untouched canal areas, canal transportation, and centering ability of WaveOne Gold, R-Motion, and Reciproc Blue systems with different apical sizes and tapers using micro-computed tomographic (micro-CT) analysis.

**Materials and Methods:**

Thirty mesial roots of mandibular molars with single curvature were selected and assigned to three groups ( *n* = 10): WaveOne Gold (25/.07v and 35/.06v), R-Motion (25/.06 and 40/.04), and Reciproc Blue (25/.08v and 40/.06v). All canals were prepared in two sequential stages with small and then larger instruments of the same system. Micro-CT scans were performed before, between, and after instrumentation. Reconstructed images (NRecon v.1.6.10, Bruker) were used to assess untouched canal walls, canal transportation, and centering ratio at 3, 5, and 7 mm from the apex.

**Statistical Analysis:**

Shapiro–Wilk test was used for normality. One-way ANOVA with Tukey's post hoc test and paired *t* -tests were applied ( *α* = 5%).

**Results:**

Preoperative canal volume and surface area showed no significant differences among groups ( *p* > 0.05). All systems demonstrated similar shaping performance regarding untouched areas, transportation, and centering ability in both preparation stages ( *p* > 0.05). A significant reduction in untouched canal walls was observed after the second preparation stage in all groups ( *p* < 0.05).

**Conclusions:**

Despite similar shaping outcomes among systems, the second preparation stage significantly reduced untouched canal walls, supporting its potential role in improving root canal cleaning efficacy.

## Introduction

Root canal treatment is a key procedure in endodontics, aiming to eliminate infection within the root canal system and reduce the risk of reinfection. A critical step is chemomechanical preparation, which shapes the root canals to enable effective irrigation, debris removal, and proper filling. 1 2 This procedure should enlarge the root canal sufficiently for disinfection while preserving tooth structure.

The introduction of NiTi instruments revolutionized endodontics by improving flexibility, fatigue resistance, and shaping performance. 3 4 5 More recently, heat-treated reciprocating systems such as Reciproc Blue, R-Motion, and WaveOne gold have enhanced clinical safety and efficiency, particularly in complex anatomies. 6 7 8 Despite these innovations, several studies demonstrate that a substantial portion of canal walls remains untouched after preparation, harboring microorganisms and debris that may compromise disinfection. 9 10

One proposed strategy to overcome this limitation is sequential preparation with a second, larger instrument from the same reciprocating system, which may increase dentin wall contact without compromising root canal anatomy. 9 11 12 However, limited evidence is available regarding its impact on shaping outcomes, especially in mandibular molars assessed by micro-CT. 13

Therefore, this study aimed to evaluate the shaping efficiency, untouched canal areas, canal transportation, and centering ability of three reciprocating systems at two preparation sizes: WaveOne Gold (25/.07v and 35/.06v), R-Motion (25/.06 and 40/.04), and Reciproc Blue (25/.08v and 40/.06v). The following null hypotheses were tested: (1) no significant differences would exist among the systems and (2) no significant differences would exist between preparation sizes within each system.

## Materials and Methods

### Sample Size Calculation

The research protocol was approved by the Local Ethics Committee (CAAE: 48570121.1.0000.0077). The sample size was determined based on the findings of De-Deus et al, 14 using an *α* error of 0.05 and a power of 95%, which indicated that a minimum of 10 teeth per group was necessary.

### Tooth Specimen Selection and Distribution

Extracted permanent mandibular first and second molars with fully separated roots, obtained for reasons unrelated to this study, were initially collected for analysis. Each specimen underwent morphometric assessment using a micro-CT scanner (SkyScan 1173; Bruker MicroCT, Kontich, Belgium) set to 70 kV and 114 µA, with a resolution of 14.25 µm. The scanning protocol included 360-degree rotation, 0.5-degree increments, and a camera exposure time of 7,000 ms with frame averaging of 5. A 1-mm aluminum filter was applied to minimize beam hardening artifacts. Image reconstruction was performed with NRecon v.1.6.10 software (Bruker MicroCT), using a 40% beam hardening correction, a ring artifact correction of 10, and standardized contrast limits. Cross-sectional images from the furcation to the apex were generated, resulting in 700 to 800 slices per specimen.

Based on these assessments, 30 teeth were selected, all exhibiting moderately curved (≤20 degrees) type II or IV mesial canals, as classified by Vertucci, 15 and type I or III isthmuses as described by Fan et al. 16 In Vertucci's classification, type II corresponds to two canals merging before the apex and type IV to two independent canals, 15 while Fan's type I represents a complete sheet-like connection between canals and type III a mixed pattern of complete and incomplete connections. 16 Specimens were stored in a 0.1% thymol solution at 5 °C until further processing. The 30 selected specimens were anatomically matched according to morphological characteristics and then evenly distributed into three experimental groups ( *n* = 10 per group): WaveOne Gold 25/.07v and 35/.06v, R-Motion 25/.06 and 40/.04, and Reciproc Blue 25/.08v and 40/.06v. Following the assessment of data normality ( *p* > 0.05, Shapiro–Wilk test), the extent of homogeneity among the groups, in terms of volume and surface area, was statistically validated ( *p* > 0.05, one-way analysis of variance [ANOVA]).

### Root Canal Preparation

After completing access cavity preparation, apical patency was confirmed by advancing a size 10 K-file (Dentsply Maillefer) until its tip was visible at the apical foramen. The silicone stop was then positioned at a reference point, establishing the working length (WL) as 1.0 mm short of this measurement. To simulate apical gas entrapment in a closed system, 17 apices were occluded with thermoplastic adhesive and sealed in polyvinylsiloxane. Root canal shaping procedures were performed by a single operator using the X-Smart Plus endodontic motor (Dentsply Sirona) set to the preset reciprocation modes recommended by each manufacturer (Reciproc, WaveOne Gold, or R-Motion). The motor operates in reciprocating motion with specific angles and speeds defined by each system's algorithm, providing consistent torque and motion patterns for each file.

The root canals were irrigated with 2 mL of 2.5% sodium hypochlorite (NaOCl), for 45 seconds, using a 30-G side-vented needle (Ultradent Products Inc, South Jordan, Utah, United States) before glide path creation, which was achieved with a size 15 K-file (Dentsply Maillefer) in all groups. Initial canal preparation was performed using the WaveOne Gold Primary (25/.07v), R-Motion 25 (25/.06), or Reciproc Blue R25 (25/.08v) instruments ( Fig. 1 ). Each instrument was applied in three in-and-out pecking motions of 2 to 3 mm of amplitude with gentle apical pressure. After three pecking motions, completing a full cycle, the instruments were removed and cleaned with sterile gauze. The canals were then irrigated with an additional 3 mL of 2.5% NaOCl for 60 seconds, and apical patency was confirmed throughout the procedure using a size 10 K-file (Dentsply Maillefer). These steps were repeated until the respective instrument reached the WL for each sample. A total of 12 mL of 2.5% NaOCl per canal was used for irrigation, followed by a final rinse of 5 mL of 17% EDTA and 5 mL of distilled water using a 30-G NaviTip needle (Ultradent) positioned 1 mm from the WL. Upon completing the initial preparation (1st Prep), the specimens underwent a postoperative micro-CT scan using the same parameters described previously.

**Fig. 1:**
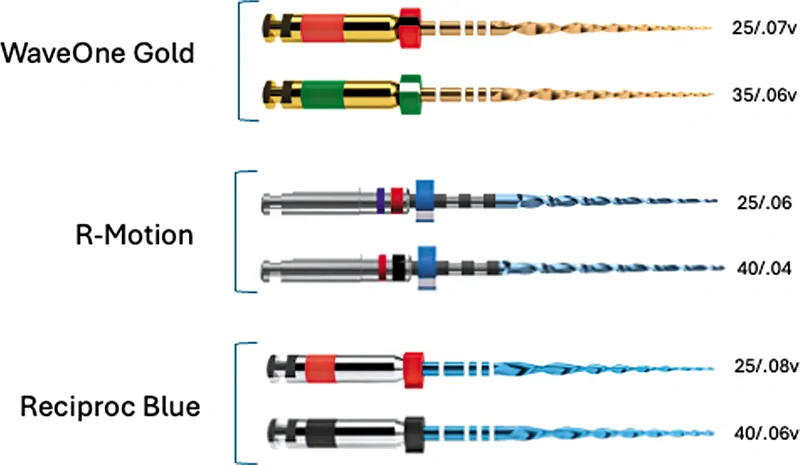
Representative illustration of the reciprocating endodontic instruments evaluated in the study, showing the different systems and sizes: WaveOne Gold (25/.07v and 35/.06v), R-Motion (25/.06 and 40/.04), and Reciproc Blue (25/.08v and 40/.06v).

Following the initial preparation stage and postoperative micro-CT scan, each sample underwent a secondary preparation stage using the WaveOne Gold Medium (35/.06v), R-Motion 40 (40/.04), or Reciproc Blue R40 (40/.06v) instruments. This second stage followed the same procedural protocols as the first, including pecking motions, irrigation steps, and verification of apical patency. Upon completing the secondary preparation (second preparation), each specimen underwent an additional postoperative micro-CT scan using the previously described parameters to document the canal modifications.

### Micro-CT Evaluation

The reconstructed images (NRecon v.1.6.10; Bruker MicroCT) were used to superimpose pre- and post-instrumentation scans. Although the mounting procedure ensured nearly identical repositioning of specimens during both scanning sessions, postoperative 3D models of roots and canals were rendered with CTAn (Bruker MicroCT) and co-registered with their respective preoperative datasets using the affine registration algorithm in 3D Slicer ( www.slicer.org ), ensuring accurate superimposition of images for analysis. 18 The percentage of untouched areas was assessed by determining the number of static voxels (voxels that remain in the same position on the canal surface both before and after instrumentation). 14 The untouched area was calculated as a percentage of the total voxels on the canal surface, using the following formula: (number of static voxels × 100)/total number of static voxels. 19 20

Canal transportation and centering ability assessments were conducted at three cross-sectional levels, located at 3, 5, and 7 mm from the apical end of each root, 21 employing the following equations:

Degree of canal transportation = (m1 − m2) − (d1 − d2),

where m1 represents the minimal distance from the mesial margin of the root to the mesial margin of the non-instrumented canal, m2 indicates the minimal distance from the mesial margin of the root to the mesial margin of the instrumented canal, d1 denotes the minimal distance from the distal margin of the root to the distal margin of the non-instrumented canal, and d2 reflects the minimal distance from the distal margin of the root to the distal margin of the instrumented canal 21 ( Fig. 2 ).

**Fig. 2:**
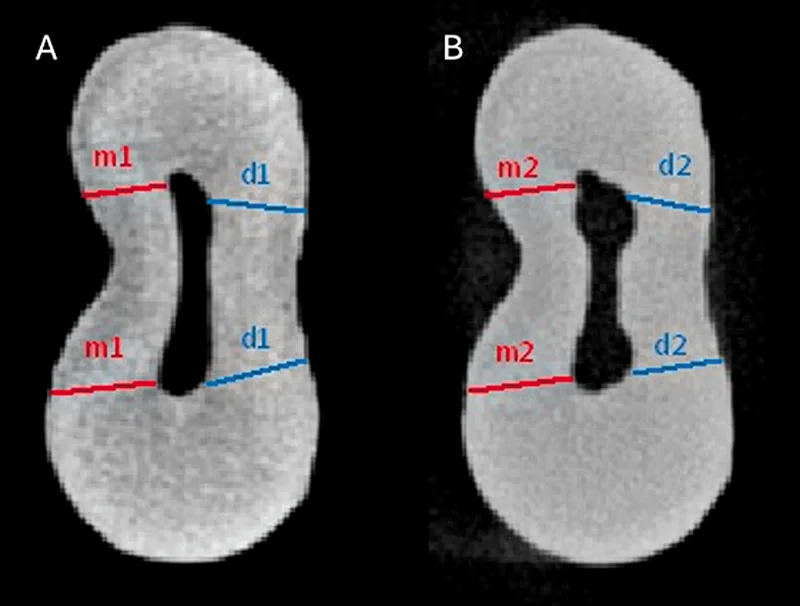
Illustration of pre-instrumentation ( **A** ) and post-instrumentation ( **B** ) measurements of the mesial and distal dentin walls.

Measurements conducted prior to and after instrumentation of the mesial root canals were executed using the ImageJ software version 1.49n (Fiji, Madison, Wisconsin, United States).

### Statistical Analysis

The data distribution was evaluated and confirmed as normal by the Shapiro-Wilk test ( *p* > 0.05). Group homogeneity was ensured based on pre-instrumentation canal volume (mm ^3^ ) and surface area (mm ^2^ ) with statistical validation following data normality assessment ( *p* > 0.05, Shapiro–Wilk test) and two-way analysis of variance ( *p* > 0.05).

Data are presented as mean ± standard deviation. Mean values were statistically compared using ANOVA to assess intergroup differences, followed by Tukey's post hoc test for multiple comparisons. Intragroup differences were assessed with a paired *t* -test. Statistical significance was set at *α* = 5%.

## Results

### Intergroup Comparisons

No statistically significant differences were observed among the systems in preoperative canal volume and surface area (Reciproc Blue: 66.7 ± 19.0 mm ^2^ /7.7 ± 2.6 mm ^3^ ; R-Motion: 65.0 ± 24.5 mm ^2^ /7.7 ± 3.0 mm ^3^ ; WaveOne Gold: 58.8 ± 16.8 mm ^2^ /6.8 ± 2.2 mm ^3^ ; *p* > 0.05). Similarly, after both the first and second preparation stages, all groups showed comparable results for canal area, volume, untouched canal walls, canal transportation, and centering ratio, with no statistically significant intergroup differences ( *p* > 0.05; Table 1 and Table 2 , Fig. 3 ).

**Table 1:** Mean and standard deviation of surface area (mm ^2^ ), volume (mm ^3^ ), and unprepared canal walls (%) of before and after preparation using the Reciproc Blue, R-Motion, and WaveOne Gold systems

Parameters		Reciproc Blue	R-Motion	WaveOne Gold
Area (mm ^2^ )	Initial	66.7 ± 19.0 ^A^	65.0 ± 24.5 ^A^	58.8 ± 16.8 ^A^
	After 1st Prep	69.4 ± 19.1 ^A^	66.6 ± 25.1 ^A^	62.2 ± 16.3 ^A^
	After 2nd Prep	70.9 ± 19.8 ^A^	69.2 ± 24.0 ^A^	63.2 ± 18.1 ^A^
Volume (mm ^3^ )	Initial	7.7 ± 2.6 ^A^	7.7 ± 3.0 ^A^	6.8 ± 2.2 ^A^
	After 1st Prep	9.1 ± 2.6 ^A^	8.7 ± 2.9 ^A^	7.9 ± 2.6 ^A^
	After 2nd Prep	10.1 ± 3.1 ^A^	9.7 ± 2.5 ^A^	8.6 ± 2.6 ^A^
Unprepared area (%)	After 1st Prep	58.9 ± 11.1 ^A^	63.4 ± 9.9 ^A^	57.9 ± 10.0 ^A^
	After 2nd Prep	46.9 ± 17.6 ^B^	49.3 ± 18.4 ^B^	50.2 ± 9.1 ^B^

Notes: Different superscript uppercase letters indicate significant differences between the same instrument and the different preparation sizes ( *p* < 0.05). No statistical differences were observed between the different tested instruments across any of the evaluated parameters ( *p* > 0.05).

**Table 2:** Mean and standard deviation of canal transportation (mm) and centering ratio values for all groups after the first and second preparation

Level/Canal		Assessment	Reciproc Blue	R-Motion	WaveOne Gold
3 mm/Buccal	1st Prep	Transportation	0.02 ± 0.10	0.05 ± 0.07	0.02 ± 0.07
		Centering ratio	0.42 ± 0.19	0.47 ± 0.29	0.69 ± 0.35
	2nd Prep	Transportation	0.04 ± 0.13	0.05 ± 0.09	0.05 ± 0.12
		Centering ratio	0.52 ± 0.29	0.55 ± 0.26	0.51 ± 0.38
3 mm/Lingual	1st Prep	Transportation	0.03 ± 0.07	0.03 ± 0.09	0.02 ± 0.08
		Centering ratio	0.50 ± 0.34	0.36 ± 0.27	0.27 ± 0.14
	2nd Prep	Transportation	−0.01 ± 0.10	0.03 ± 0.10	0.02 ± 0.08
		Centering ratio	0.54 ± 0.37	0.54 ± 0.29	0.33 ± 0.19
5 mm/Buccal	1st Prep	Transportation	−0.02 ± 0.07	−0.01 ± 0.12	−0.03 ± 0.07
		Centering ratio	0.64 ± 0.30	0.38 ± 0.33	0.54 ± 0.32
	2nd Prep	Transportation	−0.02 ± 0.07	−0.01 ± 0.14	−0.08 ± 0.11
		Centering ratio	0.62 ± 0.32	0.44 ± 0.27	0.47 ± 0.30
5 mm/Lingual	1st Prep	Transportation	0.01 ± 0.10	0.05 ± 0.09	−0.01 ± 0.11
		Centering ratio	0.66 ± 0.32	0.59 ± 0.34	0.53 ± 0.22
	2nd Prep	Transportation	−0.01 ± 0.14	0.11 ± 0.11	−0.05 ± 0.10
		Centering ratio	0.62 ± 0.32	0.61 ± 0.32	0.48 ± 0.17
7 mm/Buccal	1st Prep	Transportation	−0.07 ± 0.15	−0.02 ± 0.06	−0.10 ± 0.11
		Centering ratio	0.50 ± 0.40	0.42 ± 0.34	0.34 ± 0.21
	2nd Prep	Transportation	−0.11 ± 0.18	−0.10 ± 0.12	−0.14 ± 0.10
		Centering ratio	0.50 ± 0.28	0.42 ± 0.33	0.42 ± 0.33
7 mm/Lingual	1st Prep	Transportation	−0.09 ± 0.14	−0.003 ± 0.10	−0.12 ± 0.11
		Centering ratio	0.51 ± 0.31	0.54 ± 0.38	0.47 ± 0.23
	2nd Prep	Transportation	−0.08 ± 0.13	−0.01 ± 0.11	−0.14 ± 0.14
		Centering ratio	0.57 ± 0.29	0.53 ± 0.29	0.41 ± 0.19

Note: No statistical differences were observed between the different tested instruments and different preparation steps across any of the evaluated parameters ( *p* > 0.05).

**Fig. 3:**
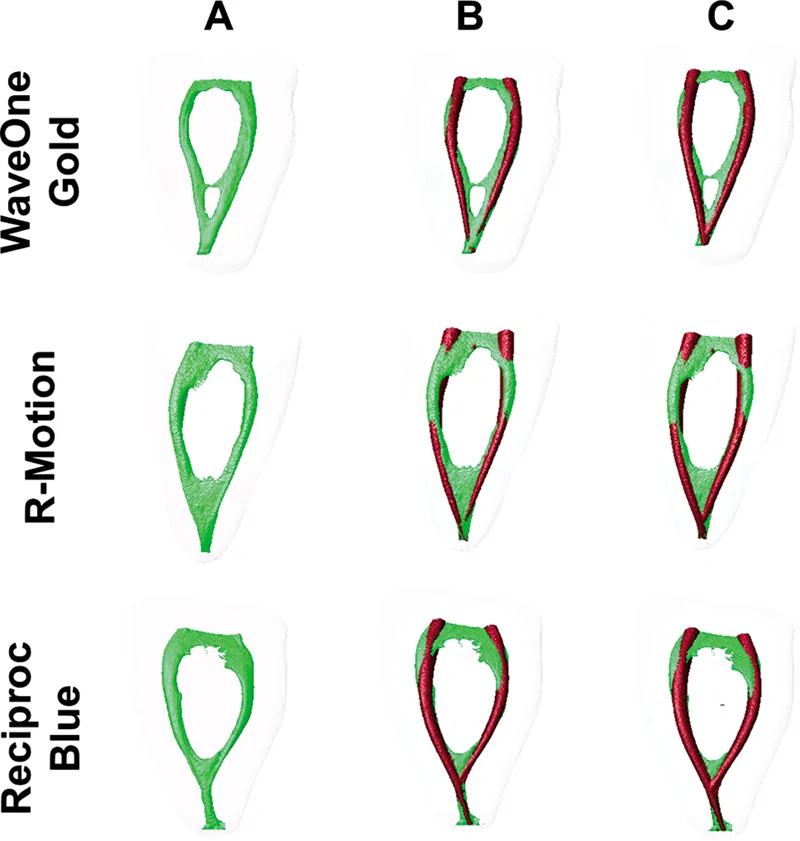
Representative three-dimensional images of root canals before ( **A** column—green), after first preparation ( **B** column—red), and second preparation ( **C** column—red) with the systems: WaveOne Gold, R-Motion, and Reciproc Blue.

### Intragroup Comparisons

When baseline, first, and second preparation stages were compared within each system, no significant differences were found for canal area, volume, transportation, or centering ratio ( *p* > 0.05) ( Table 1 and Table 2 ). However, all systems demonstrated a significant reduction in untouched canal walls after the second preparation stage: from 58.9 to 46.9% for Reciproc Blue, from 63.4 to 49.3% for R-Motion, and from 57.9 to 50.2% for WaveOne Gold ( *p* < 0.05; Table 1 , Fig. 3 ).

## Discussion

The rationale for performing a second preparation stage is based on evidence that single-file techniques often leave a substantial portion of the canal walls untouched, even with modern heat-treated instruments. Previous studies have suggested that additional enlargement, particularly when performed with a second instrument of the same system, may enhance dentin wall contact and improve disinfection. 9 22 However, evidence supporting this approach remains scarce, especially in mandibular molars, and clinical validation is needed. The present study addresses this gap by providing micro-CT evidence on shaping outcomes following sequential instrumentation, comparing the centering ability, canal transportation, and untouched areas of three reciprocating systems (WaveOne Gold, R-Motion, and Reciproc Blue) at both the initial and second preparation stages.

The results showed that all systems performed similarly in terms of canal transportation, centering ability, and untouched canal walls; therefore, the first null hypothesis tested was accepted. These findings are consistent with previous studies reporting comparable performance among reciprocating systems in shaping curved canals, likely due to similarities in tip and taper sizes, blade design, and instrument flexibility. 20 23 24 Such characteristics, enhanced by advances in metallurgy, thermal treatments, and manufacturing processes, explain the consistent shaping outcomes and reflect a high level of refinement achieved by these instruments, which are capable of addressing anatomical complexities in routine clinical practice.

The percentage of untouched canal walls is clinically relevant because these areas may harbor microorganisms and tissue remnants, potentially compromising disinfection and negatively affecting endodontic prognosis. 9 In this study, quantitative analysis revealed a significant reduction in untouched walls after the second preparation stage in all groups, leading to the rejection of the second null hypothesis. Although the systems share similar overall performance, their distinct alloy treatments, cross-sectional geometries, and taper configurations justify further comparison, especially in multistage approaches. Our findings underscore the benefit of sequential instrumentation in increasing dentin wall coverage, corroborating previous reports that highlight the importance of supplemental preparation for improving the removal of pulp tissue and bacterial debris, particularly in the mesial canals of mandibular molars. 9 18 25 26 Nevertheless, untouched areas persisted, confirming the inherent limitation of mechanical instruments in flattened or oval-shaped canals. 14 18 27 Addressing these limitations requires further innovations in instrument design and complementary strategies, such as enhanced irrigation or adjunctive technologies to achieve more comprehensive canal cleaning.

In assessing shaping outcomes, canal transportation is another critical parameter, as excessive enlargement may compromise tooth structure. 28 Previous studies have shown that rotary system characteristics, including taper, cross-sectional design, and kinematics, influence volumetric changes and transportation in curved canals. İslam et al 29 reported that ProTaper Gold produced the greatest transportation, while R-Motion and RaceEvo better preserved canal anatomy, a performance attributed to R-Motion's thinner core, higher flexibility, and unique cross-sectional design. Similarly, Mustafa 30 found that R-Motion promoted the lowest transportation and best centering ability in mandibular molars, outperforming Reciproc Blue and ProTaper Gold, while also reducing untouched areas. De Carvalho et al 31 confirmed that thermally treated NiTi instruments maintained canal anatomy with minimal transportation, highlighting the role of metallurgy in shaping safety. Based on these reports, one might expect instruments with different apical sizes and tapers to produce different outcomes. However, no significant differences were found in the present study. This contrasts with Elzaurdia et al, 32 who observed greater coronal transportation with Reciproc Blue compared with R-Motion, but agrees with Alsulaiman et al, 24 who reported shaping ability between R-Motion and WaveOne Gold, with R-Motion causing less dentin removal and transportation. Taken together, these findings suggest that modern heat-treated reciprocating systems, supported by features such as regressive taper and reciprocating motion, provide conservative shaping while minimizing anatomical deviations, even when larger tapers are used.

The regressive taper design, which progressively reduces instrument diameter along its length, likely contributed to the comparable results among systems. This geometry limits coronal dentin removal, reduces stress concentration in critical areas, and favors conservative shaping while maintaining adequate apical enlargement. Combined with reciprocating motion, it ensures controlled engagement of canal walls, decreases torsional stress, and enhances safety in complex anatomies.

Despite its strengths, this study has certain limitations. The in vitro design cannot fully replicate the clinical conditions, where patient and operator-related factors may influence outcomes. Moreover, while micro-CT provided precise data on canal morphology and preparation, it does not reflect the chemical action of irrigants against biofilm in untouched areas. The focus on mandibular molars, although clinically justified, also restricts extrapolation to other tooth types with different morphology. Still, the study presents several strengths, including the use of micro-CT as a nondestructive tool that allowed anatomical pairing and reduced variability. This pairing, confirmed by the preoperative differences in canal morphology, minimized anatomical bias and strengthened group comparability. 33 In addition, the use of mandibular molars provided a clinically relevant model, given their complex mesial canal anatomy with concavities and irregularities that challenge instrumentation. 34 35 36 37 By integrating rigorous methodology, advanced imaging, and clinically relevant samples, the study offers important insights into the shaping ability and limitations of reciprocating systems.

Our findings demonstrate that different reciprocating systems with varying tapers and apical sizes can achieve comparable shaping outcomes. This reinforces their safe application in clinical practice and highlights the importance of features, such as regressive taper and enhanced flexibility in preserving canal anatomy and minimizing errors. The ability to obtain consistent results across systems also underscores their adaptability in diverse scenarios, offering clinicians reliable options for endodontic treatment in anatomically complex cases.

Finally, the results should be interpreted with caution. Excessive enlargement of the apical third may lead to unnecessary dentin removal, increasing the risk of transportation, perforation, or vertical root fracture, 34 38 without necessarily improving the removal of infected tissue. 39 Thus, apical enlargement should be sufficient to allow irrigation and disinfection while preserving root structure. The regressive taper design represents a practical solution, allowing larger apical diameters while limiting dentin sacrifice coronally, thereby balancing safety and efficacy. and preserving root strength. Adjunctive antibacterial strategies, such as ultrasonic or subsonic agitation of irrigants, 40 GentleWave, 41 or laser-based methods, may further optimize cleaning, although their clinical effectiveness has yet to be fully demonstrated. Future studies should assess the applicability of these findings in other tooth types with complex anatomies and explore whether combining sequential instrumentation with advanced irrigation techniques can improve the management of untouched areas.

## Conclusion

In conclusion, WaveOne Gold, R-Motion, and Reciproc Blue performed similarly in shaping ability. The second preparation stage reduced untouched canal walls, and systems with regressive tapers appear to balance apical enlargement with dentin preservation. However, as untouched areas remained, adjunctive irrigation techniques are still required for optimal disinfection.
